# SIX4 upregulates IDH1 and metabolic reprogramming to promote osteosarcoma progression

**DOI:** 10.1111/jcmm.17650

**Published:** 2023-01-04

**Authors:** Bing Li, Xiaoqian Dang, Jiafeng Duan, Guangyang Zhang, Jia Zhang, Qichun Song

**Affiliations:** ^1^ Department of Orthopaedics The Second Affiliated Hospital of Xi'an Jiaotong University Xi'an China; ^2^ Department of Orthopaedics, Xi'an No.3 Hospital The Affiliated Hospital of Northwest University Xi'an China; ^3^ Department of Implant, Nobel Stomatology Hospital Xi'an China; ^4^ Key Laboratory of Shaanxi Province for Craniofacial Precision Medicine Research, College of Stomatology Xi'an Jiaotong University Xi'an China

**Keywords:** glycolysis, IDH1, osteosarcoma, SIX4

## Abstract

Metabolism reprogramming plays an important role in tumorigenesis and osteosarcoma metastasis. Sine oculis homeobox 4 (SIX4) is reported to be a key transcription factor that is involved in glycolysis reprogramming of cancer cells. However, the role of SIX4 in osteosarcoma progression remains unknown. The expression profile of SIX4 in OS was evaluated in surgery samples of osteosarcoma patients. Functional studies were performed in vitro and in vivo. We found that SIX4 is significantly overexpressed in osteosarcoma and related to the undesirable prognosis of osteosarcoma patients. SIX4 promotes progression of osteosarcoma via upregulating isocitrate dehydrogenase 1 (IDH1), which provides novel prognostic biomarkers and promising therapeutic targets for osteosarcoma patients.

## 1 INTRODUCTION

Osteosarcoma is one of the deadly primary bone malignancies worldwide, and most patients with osteosarcoma have limited treatment options.^1^, ^2^ Although tremendous approaches have been made, the outcomes of osteosarcoma patients remain unsatisfactory.^3^, ^4^ Moreover, osteosarcoma is a highly invasive tumour with frequent distant metastasis and recurrence.^5^, ^6^ Therefore, an urgent need to develop molecular targeted agents with high osteosarcoma specificity is required.

The sine oculis homeobox 4 (SIX4) transcription factor, which belongs to the superfamily of homeobox gene family, has been identified contributes to tumour initiation, progression and metastasis.^7^ For example, upregulation of SIX4 promotes tumour growth or metastasis in oesophageal squamous cell carcinoma, hepatocellular carcinoma and breast cancer.^8^, ^9^, ^10^ These findings indicate that deregulation of SIX4 plays critical roles in cancer progression and metastasis. However, the expression and functional role of SIX4 in osteosarcoma remains unknown.

In this study, we demonstrated that overexpression of SIX4 promoted osteosarcoma metastasis by changing glycolysis and upregulating isocitrate dehydrogenase 1(IDH1) expression. Our work provides novel prognostic biomarkers and promising therapeutic targets for osteosarcoma patients.

## 2 MATERIALS AND METHODS

### 2.1 Osteosarcoma specimens

A total of 24 cases of osteosarcoma tissues and the adjacent non‐tumour tissues were obtained from patients who underwent surgical excision at the Second Affiliated Hospital of Xi'an Jiaotong University, Xi'an No.3 Hospital, the Affiliated Hospital of Northwest University, Shaanxi, China. All patients were obtained informed consent, and this study was approved by the ethics committee of the Second Affiliated Hospital of Xi'an Jiaotong University.

### 2.2 Cell lines and cell treatment

The human osteosarcoma cell lines U2OS, 143B, MNNGHOS, MG63, G292 and the normal osteoblast cell line hFOB1.19 were cultured in Dulbecco's modified Eagle's medium (DMEM) with 10% fetal bovine serum (FBS), penicillin (100 U/ml) and streptomycin (100 ng/ml) at 37°C with 5% CO_2_. U2OS cells were transfected with pBabe‐SIX4 to generate SIX4 overexpression cells, while MG63‐*sh*SIX4 cells were established by lentivirus infections with pLKO.1‐*sh*SIX4.

### 2.3 Western blot

Osteosarcoma tissues and cell lines were collected and lysed in RIPA buffer with protease inhibitor (1:400, Millipore, USA) for 30 mins, followed by centrifugation for 15 min at 12,000 rpm at 4°C. The supernatants were collected, and the protein concentration was quantified by using BCA assay kit (Beyotime Biotechnology). A total of 30 μg of protein were separated by SDS‐PAGE gels and transferred to polyvinylidene difluoride membranes. The membranes were blocked before being incubated with primary antibodies: anti‐SIX4 antibody (abcam, ab176713, 1:500); anti‐IDH1 antibody (abcam, ab172964, 1:1000); anti‐β‐actin antibody (abcam, ab6276, 1:5000). The corresponding horseradish peroxidase‐conjugated secondary antibodies were used, and the proteins were detected using chemiluminescent detection reagents and films.

### 2.4 Transwell assay

Transfected osteosarcoma cells were cultured at 1 × 10^4^ cells/200 μl in Transwell® cell culture insert (8 μm pore size, Corning, USA) and allowed to migrate for 24 h. Then, the chamber was fixed with methanol and stained with crystal violet (0.1%) for 15 min. The cells on the upper surface of the filters were removed, and the cells that migrated through the filters were counted.

### 2.5 Wound healing assay

Transfected osteosarcoma cells were seeded in a 12‐well plate with 80% of confluency. Vertical and horizontal lines were drawn on the bottom of each well by a sterile pipette tip and cultured for 24 hours. The same view of the scratch was captured, and the migration rate was evaluated by Image J software.

### 2.6 Gene expression analysis using real‐time PCR

Osteosarcoma tissues and cell lines were collected, and the total RNA was extracted using TRIzol (Invitrogen) according to the manufacturer's instructions. Real‐time PCR was done with SYBR Green Master mix (Takara). The 2^−ΔΔ*C*t^ method was employed to analyse the gene expression.

### 2.7 Luciferase reporter assay

Luciferase activity was detected using the Dual Luciferase Assay (Promega) according to the manufacturer's instructions. The transfected cells were lysed and centrifuged at maximum speed for 1 min. Relative luciferase activity was determined using a ModulusTM TD20/20 Luminometer (Turner Biosystems), and the transfection efficiencies were normalized according to the Renilla activity.

### 2.8 Statistical analyses

Student's *t*‐test was used to compare individual data between two groups. One‐way anova test was used to compare the data among three or more groups. Pearson's correlation coefficient was calculated to show the relationship between the expression levels of SIX4 and IDH1 mRNA. The Kaplan–Meier method and the log‐rank test were used to compare patient survival. Data are presented as mean ± SD or median with interquartile range. *p* < 0.05 was considered statistical significance.

## 3 RESULTS

### 3.1 Upregulation of SIX4 in osteosarcoma is important for metastasis and poor prognosis

**FIGURE 1:**
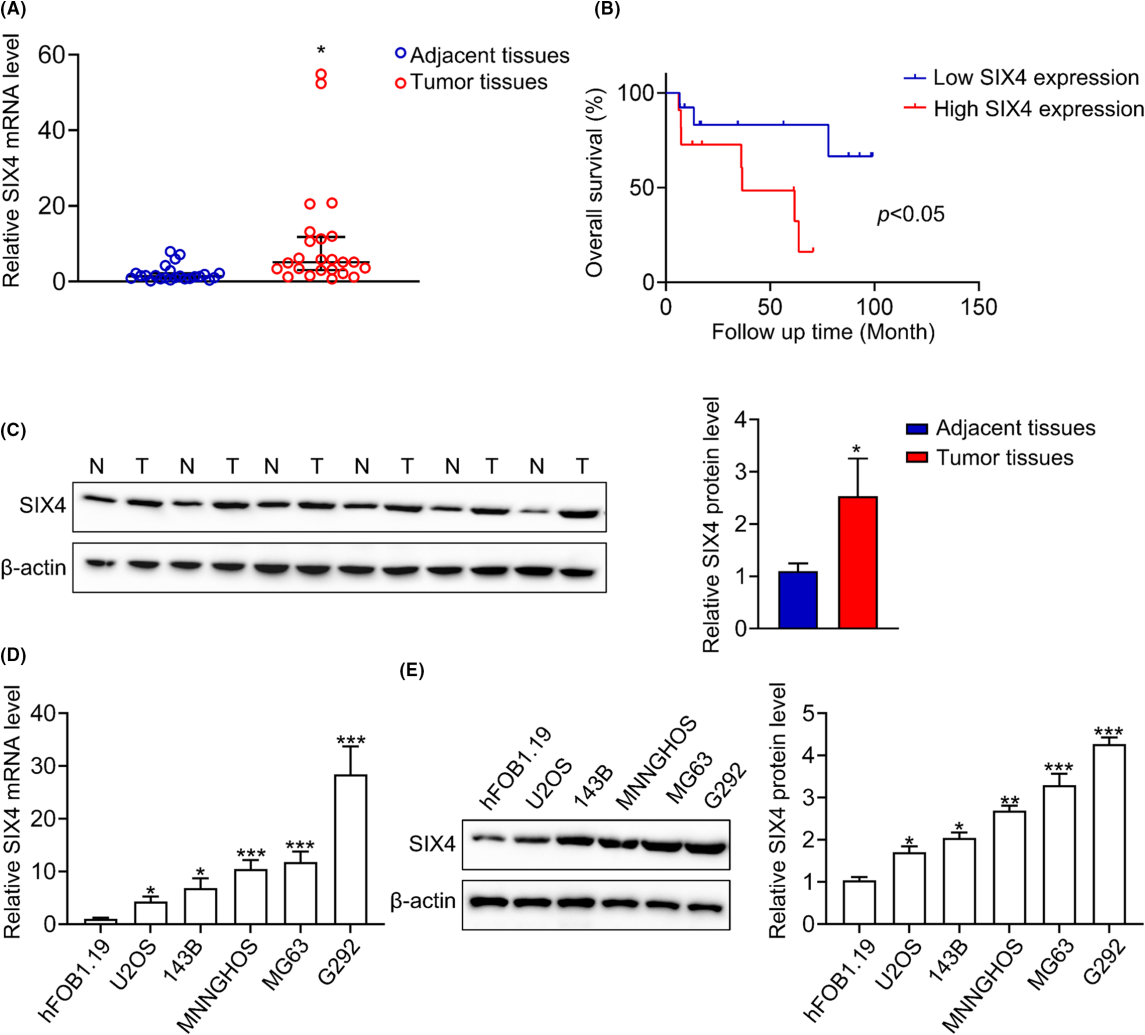
Upregulation of SIX4 in osteosarcoma is important for metastasis and poor prognosis. (A) PCR analysis of SIX4 mRNA expression in osteosarcoma samples. Data are mean ± SD, *n* = 24. *p < 0.05. (B) Overall survival of osteosarcoma patients with different SIX4 levels. (C) The expression of SIX4 in osteosarcoma tissues and adjacent tissues by Western blot. Data are mean ± SD, *p < 0.05. (D) PCR analysis of SIX4 mRNA expression in osteosarcoma cell lines. Data are mean ± SD, *p < 0.05, ***p < 0.001. (E) Western blot analysis of SIX4 protein expression in osteosarcoma cell lines. Data are mean ± SD, *p < 0.05, ***p < 0.001.

The upregulation of SIX4 mRNA and protein was further validated in fresh samples from surgery patients of osteosarcoma (Figure 1A,C). More importantly, higher SIX4 level predicts poorer outcomes of osteosarcoma patients (Figure 1B). To better understand the role of SIX4 in osteosarcoma, we evaluated the SIX4 expression in human osteosarcoma cell lines (U2OS, 143B, MNNGHOS, MG63, G292), as well as bone cell line (hFOB1.19). Consistently, the SIX4 mRNA and protein expression were upregulated in osteosarcoma cell lines compared with the bone cell line hFOB1.19 (Figure 1D,E). In summary, combined with the public data and our results, we demonstrated that upregulation of SIX4 in osteosarcoma is important for metastasis and poor prognosis.

### 3.2 SIX4 is important for the migration of osteosarcoma cells

**FIGURE 2:**
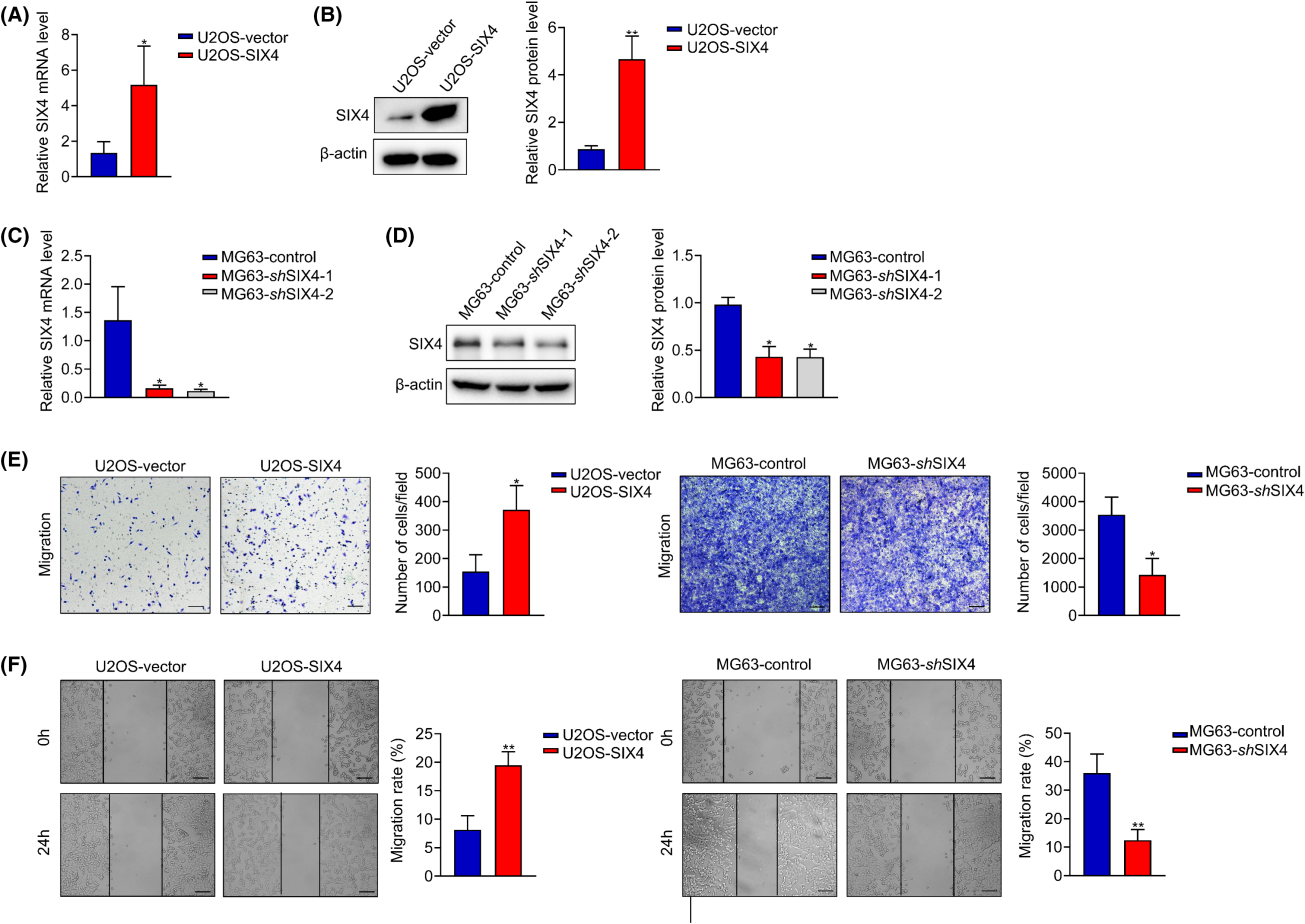
SIX4 is important for the migration of osteosarcoma cells. (A) PCR analysis of SIX4 mRNA expression in U2OS cells. Data are mean ± SD, *p < 0.05. (B) Western blot analysis of SIX4 protein expression in U2OS cells. Data are mean ± SD, **p < 0.01. (C) PCR analysis of SIX4 mRNA expression in MG63 cells. Data are mean ± SD, *p < 0.05. (D) Western blot analysis of SIX4 protein expression in MG63 cells. Data are mean ± SD, *p < 0.05. (E) Transwell assay of osteosarcoma cells with SIX4 overexpression or downregulation. Scale bars, 100 μm. Data are mean ± SD, *p < 0.05. (F) Wound healing assay of osteosarcoma cells with SIX4 overexpression or downregulation. Scale bars, 100 μm. Data are mean ± SD, **p < 0.01.

To investigate the function of SIX4 in osteosarcoma, human osteosarcoma cell line MG63 and U2OS were used for SIX4 knockdown and overexpression, respectively (Figure. 2A–D). SIX4 downregulation significantly inhibited cell migration in MG63 cells (Figure 2E,F), whereas stably overexpressing SIX4 promoted cell migration in U2OS cells, as indicated by wound healing and transwell assays (Figure 2E,F). Collectively, these results demonstrated that SIX4 is important for the migration of osteosarcoma cells.

### 3.3 SIX4 transcriptionally activates IDH1

**FIGURE 3:**
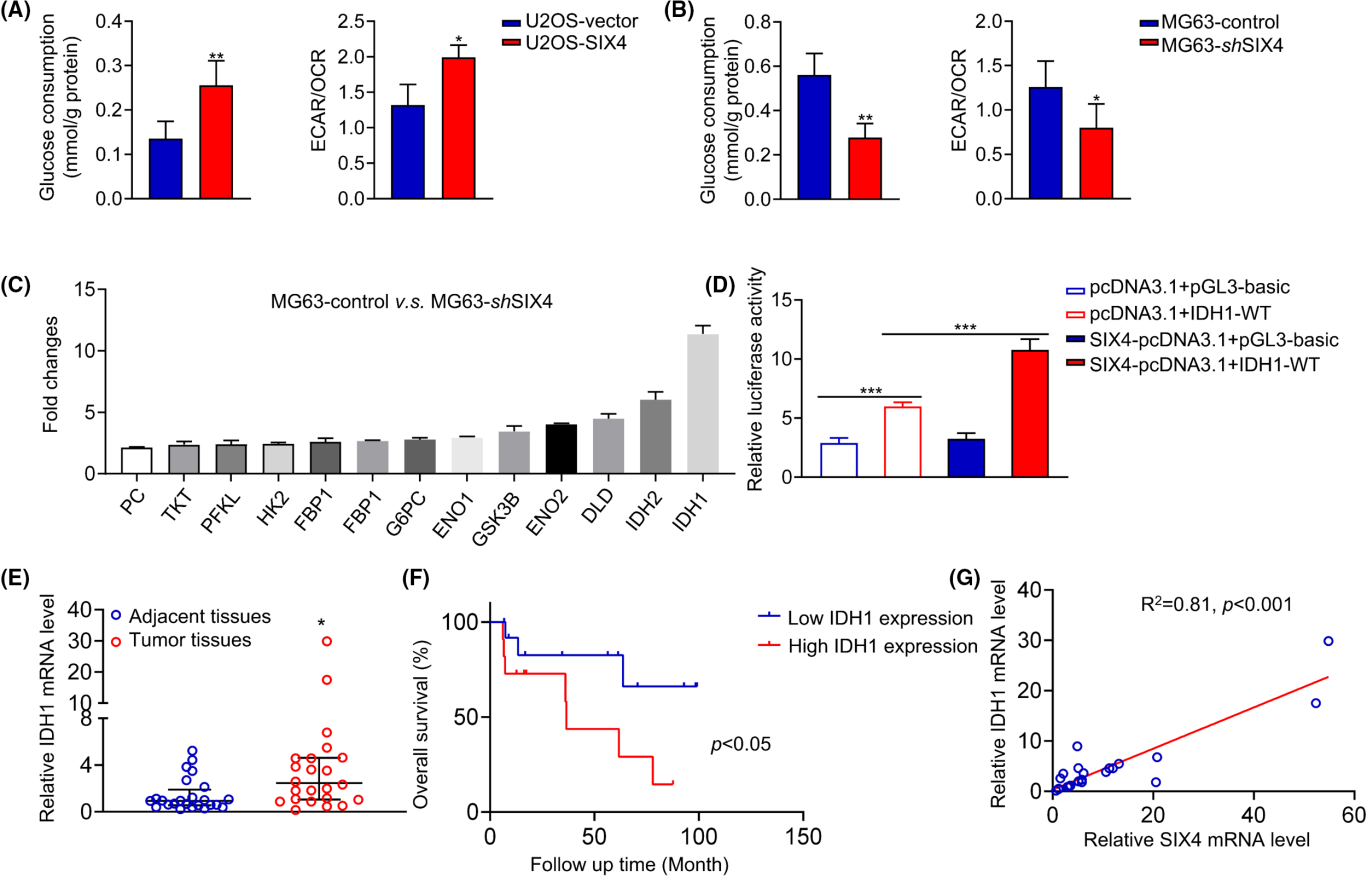
SIX4 transcriptionally activates IDH1. (A, B) ECAR/OCR and glucose consumption assay of osteosarcoma cells with SIX4 overexpression or downregulation. Data are mean ± SD, *p < 0.05, **p < 0.01. (C) Human glucose metabolism PCR array analysis of osteosarcoma cells. Data are mean ± SD, *p < 0.05. (D) Luciferase reporter assay for SIX4 transcriptionally activates IDH1. Data are mean ± SD, ***p < 0.001. (E) PCR analysis of IDH1 mRNA expression in osteosarcoma samples. Data are mean ± SD, *n* = 24. *p < 0.05. (F) Overall survival of osteosarcoma patients with different IDH1 levels. (G) Correlations between SIX4 and IDH1 expression.

Glycolysis is a key feature for tumorigenesis and metastasis, and SIX4 has been reported to be crucial for glycolysis.^11^, ^12^, ^13^ To understand whether SIX4‐mediated osteosarcoma metastasis is associated with glycolysis, we examined the extracellular acidification rate/oxygen consumption rate (ECAR/OCR). As expected, overexpression of SIX4 significantly increased ECAR/OCR, whereas downregulation of SIX4 decreased ECAR/OCR compared with the corresponding control cells (Figure 3A), suggesting that SIX4 is required for glycolysis. In addition, glucose consumption was upregulated when SIX4 is overexpressed, while the glucose consumption was decreased when SIX4 is downregulated (Figure 3B), further confirming that SIX4 is required for glycolysis. We also utilized the human glucose metabolism PCR array to gain an in‐depth understanding of SIX4 for glycolysis in osteosarcoma. The results showed that downregulation of SIX4 reduced the level of several metabolic enzymes associated with tricarboxylic acid cycle (TCA cycle), among which IDH1 decreased most significantly (Figure 3C). The luciferase reporter assay further demonstrated that SIX4 transcriptionally activates IDH1 (Figure 3D). Upregulation of IDH1 was further validated in osteosarcoma patients with undesirable outcomes (Figure 3E,F). Besides, the upregulation of SIX4 was correlated with increased expression of IDH1of osteosarcoma (Figure 3G). Altogether, upregulation of SIX4 altered glycolysis and transcriptionally activates IDH1 in osteosarcoma.

### 3.4 IDH1 knockdown significantly suppressed the SIX4‐driven metastasis of osteosarcoma

**FIGURE 4:**
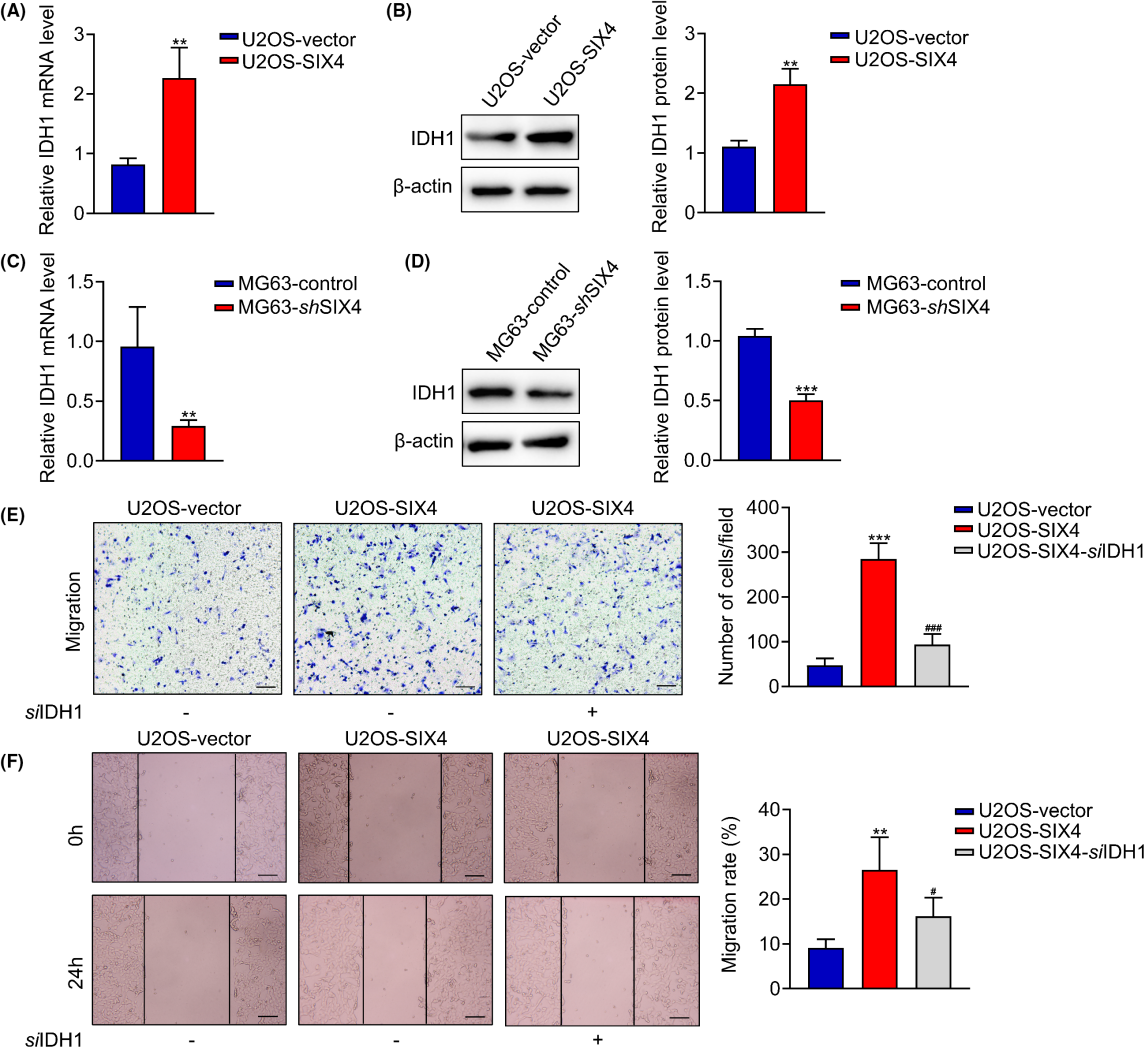
IDH1 knockdown significantly suppressed the SIX4‐driven metastasis of osteosarcoma. (A) PCR analysis of IDH1 mRNA expression in U2OS cells. Data are mean ± SD, **p < 0.01. (B) Western blot analysis of IDH1 protein expression in U2OS cells. Data are mean ± SD, **p < 0.01. (C) PCR analysis of IDH1 mRNA expression in MG63 cells. Data are mean ± SD, **p < 0.01. (D) Western blot analysis of IDH1 protein expression in MG63 cells. Data are mean ± SD, ***p < 0.001. (E) Transwell assay of osteosarcoma cells. Scale bars, 100 μm. Data are mean ± SD, ***p < 0.001 (U2OS‐vector vs. U2OS‐SIX4), ###*p* < 0.001 (U2OS‐SIX4 vs. U2OS‐SIX4‐*si*IDH1). (F) Wound healing assay of osteosarcoma cells with SIX4 overexpression or downregulation. Scale bars, 100 μm. Data are mean ± SD, **p < 0.01 (U2OS‐vector vs. U2OS‐SIX4), #*p* < 0.05 (U2OS‐SIX4 vs. U2OS‐SIX4‐*si*IDH1).

To explore whether SIX4‐mediated osteosarcoma metastasis is associated with upregulation of IDH1, we examined the expression of IDH1 in SIX4 overexpression and knockdown cells. Overexpression of SIX4 leads to increased IDH1 mRNA and protein expression, whereas downregulation of SIX4 decreased IDH1 expression compared with the corresponding control cells (Figure 4A–D), suggesting that IDH1 is involved in the pathogenesis of SIX4‐driven metastasis. Consistently, cell migration was significantly inhibited when knockdown IDH1 in SIX4 overexpressing cells, as determined by transwell and wound healing assays (Figure 4E,F). Taken together, these results demonstrated that IDH1 knockdown significantly suppressed the SIX4‐driven metastasis of osteosarcoma.

## 4 DISCUSSION

Accumulating evidence has shown that glycolysis is a key feature for tumorigenesis and metastasis of osteosarcoma.^14^, ^15^, ^16^ Sine oculis homeobox (SIX) family transcriptional factors were reported to be important for glycolysis in carcinogenesis.^17^, ^18^ SIX4 belongs to the sine oculis homeobox family that has been reported to be required for tumour progression, such as leukaemia, oesophageal squamous cell carcinoma, hepatocellular carcinoma and pancreatic ductal adenocarcinoma.^8^, ^9^, ^19^, ^20^

Given the significance of SIX4 in carcinogenesis and metabolic reprogramming, we test the expression of SIX4 in osteosarcoma tissues and cell lines. Our results showed that SIX4 was upregulated in osteosarcoma, and its upregulation predicted poor outcomes for osteosarcoma. These findings suggested that SIX4 plays an important role in osteosarcoma. However, no evidence is available to demonstrate the functional role of SIX4 in osteosarcoma. Therefore, we generated SIX4 overexpressing and knockdown cell lines to further explore the role and mechanisms of SIX4 in the pathogenesis of osteosarcoma. We found that SIX4 is required for the migration of osteosarcoma cells. Thereafter, we evaluated the ECAR/OCR and glucose consumption to figure out whether the pro‐migration capacity of SIX4 is associated with glycolysis. As expected, both ECAR/OCR and glucose consumption were upregulated when SIX4 is overexpressed, while downregulation of SIX4 spontaneously decreased the ECAR/OCR and glucose consumption of tumour cells. All these findings demonstrated that SIX4 promoted osteosarcoma progression via regulating tumour cell glycolysis. Then, we analysed serious glycolysis‐related genes and found that IDH1 can be transcriptionally regulated by SIX4 to promote cancer cell migration. IDH1 is a key enzyme of TCA cycle that catalyses the conversion of isocitrate to alpha‐ketoglutarate.^21^, ^22^ Upregulation of IDH1 has been found in osteosarcoma development and is associated with poor overall survival.^23^ We found that cell migration was significantly inhibited when knockdown IDH1 in SIX4 overexpressing cells, as determined by transwell and wound healing assays (Figure 4E,F). Taken together, these results demonstrated that IDH1 knockdown significantly suppressed the SIX4‐driven metastasis of osteosarcoma.

In conclusion, our study found that SIX4 promotes progression of osteosarcoma via upregulating IDH1 and metabolic reprogramming, which provides novel prognostic biomarkers and promising therapeutic targets for osteosarcoma patients.

## AUTHOR CONTRIBUTIONS

**Bing Li:** Investigation (lead). **Xiaoqian Dang:** Investigation (supporting); validation (supporting). **Jiafeng Duan:** Investigation (supporting); validation (supporting). **Guangyang Zhang:** Investigation (supporting); validation (supporting). **Jia Zhang:** Investigation (supporting); validation (supporting). **Qichun Song:** Conceptualization (lead); data curation (lead); supervision (lead).

## CONFLICT OF INTEREST

The authors declare no competing interests.

## Supporting information


Appendix S1

## Data Availability

Data available in article Supporting Information.
