# Supplementary material for: SIX4 upregulates IDH1 and metabolic reprogramming to promote osteosarcoma progression
Source: J Cell Mol Med. 2023 Jan 4;27(2):259–65. doi: 10.1111/jcmm.17650 (PMC9843517; doi:10.1111/jcmm.17650)

**SIX4 upregulates IDH1 and** **metabolic reprogramming to promote** **osteosarcoma progression**

Bing Li^1,2,#^, Xiaoqian Dang^1^, Jiafeng Duan^3^, Guangyang Zhang^1^, Jia Zhang^4^, Qichun Song^1,*^

^1^Department of Orthopaedics, The Second Affiliated Hospital of Xi’an Jiaotong University, Xi'an, Shaanxi, 710004, P.R. China;

^2^Department of Orthopaedics, Xi'an No.3 Hospital, the Affiliated Hospital of Northwest University, Xi' an, Shaanxi, 710018, P.R . China;

^3^Department of Head and Neck Tumor Surgery, College of Stomatology, Xi’an Jiaotong University, Xi'an, Shaanxi, 710004, P.R. China;

^4^ Department of Pathology, College of Stomatology, Xi’an Jiaotong University, Xi'an, Shaanxi, 710004, P.R. China;

^#^Co-first authors

*Corresponding author:

Qichun Song, Department of Orthopaedics, The Second Affiliated Hospital of Xi’an Jiaotong University, Xi'an, Shaanxi, 710004, P.R. China; E-mail: [qichuns_xjtu@163.com](mailto:qichuns_xjtu@163.com)

**Supplementary Materials and Methods**

**CCK8 and BrdU assay**

CCK8 assay was operated using CCK8 assay kit (Beyotime Biotechnology, China). Transfected cells were inoculated in 96-well plates and cultured for 5 days respectively. Then, 20µl CCK8 solution was added in each well and plates were incubated at 37°C for 3 hours. After removing the supernatant, 100 µl DMSO per well was added and kept shaking for 10 minutes. The optical density (OD) value was measured at 490 nm with microplate reader (Elx800, BioTek, USA). BrdU incorporating assay was performed using BrdU Cell Proliferation Assay Kit (Millipore) according to the instructions.

**Supplementary Figures**

**Supplementary Figure. 1 SIX4 promotes cell proliferation of osteosarcoma.** (A and D) Colony formation assay of osteosarcoma cells with SIX4 overexpression or downregulation; (B and E) BrdU assay of osteosarcoma cells with SIX4 overexpression or downregulation. Data are mean ± SD, **p* <0.05; (C and F) CCK8 assay of osteosarcoma cells with SIX4 overexpression or downregulation. Data are mean ± SD, **p* <0.05.


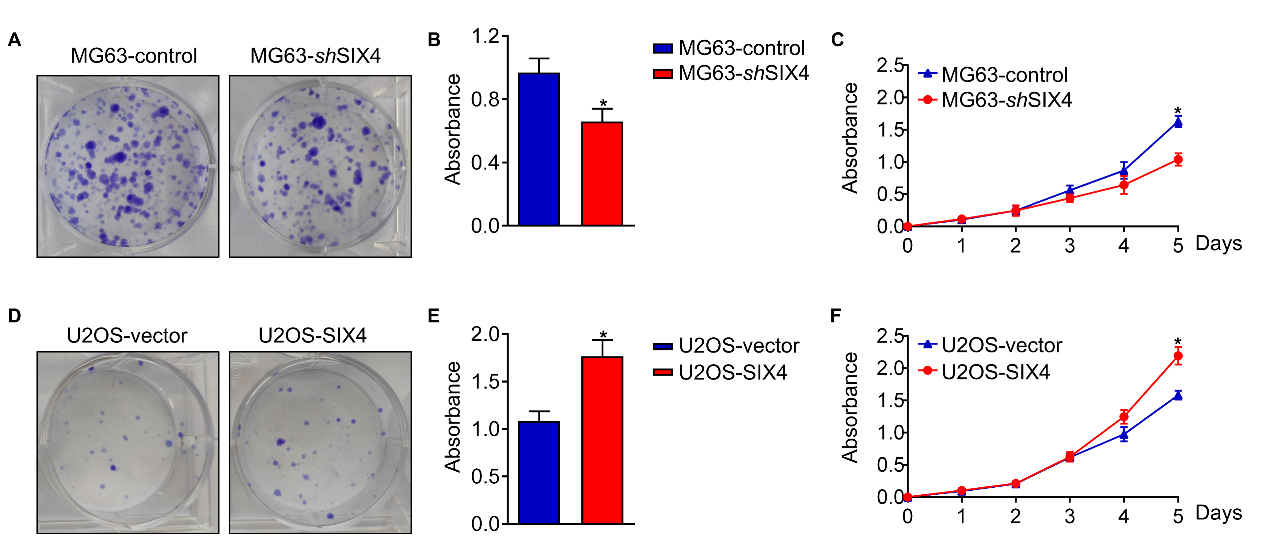


**Supplementary Figure. 2 IDH1 knockdown significantly suppressed the SIX4-driven proliferation of osteosarcoma.** (A) Colony formation assay of indicated cells; (B) BrdU assay of indicated cells. Data are mean ± SD, **p* <0.05, ***p* <0.01.


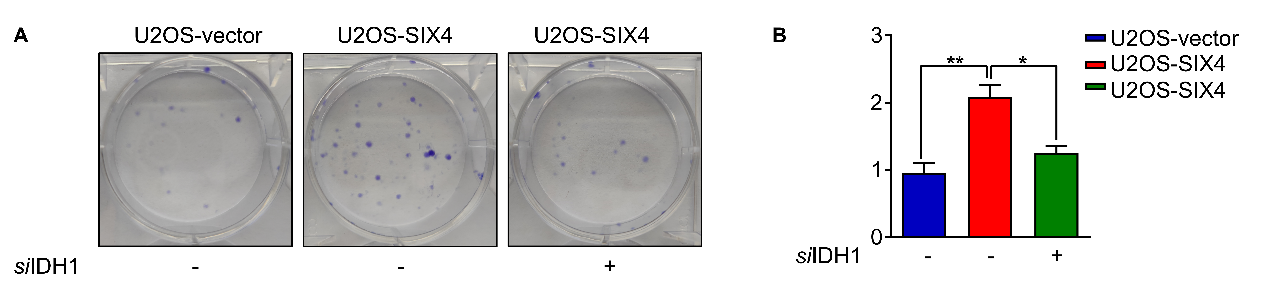

Supplement: Supplementary file 1 — Appendix S1 [file JCMM-27-259-s001.docx]
